# Spiking Characteristics of Network-Mediated Responses Arising in Direction-Selective Ganglion Cells of Rabbit and Mouse Retinas to Electric Stimulation for Retinal Prostheses

**DOI:** 10.1109/TNSRE.2021.3128878

**Published:** 2021-11-25

**Authors:** Yanjinsuren Otgondemberel, Hyeonhee Roh, Shelley I. Fried, Maesoon Im

**Affiliations:** Brain Science Institute, Korea Institute of Science and Technology (KIST), Seoul 02792, South Korea, and also with the Division of Bio-Medical Science & Technology, KIST School, University of Science and Technology (UST), Seoul 02792, South Korea.; Brain Science Institute, Korea Institute of Science and Technology (KIST), Seoul 02792, South Korea, and also with the Division of Electrical Engineering, College of Engineering, Korea University, Seoul 02841, South Korea.; VA Boston Healthcare System, Boston, MA 02130 USA, and also with the Department of Neurosurgery, Massachusetts General Hospital, Harvard Medical School, Boston, MA 02114 USA.; Department of Neurosurgery, Massachusetts General Hospital, Harvard Medical School, Boston, MA 02114 USA. He is now with the Brain Science Institute, Korea Institute of Science and Technology (KIST), Seoul 02792, South Korea, and also with the Division of Bio-Medical Science & Technology, KIST School, University of Science and Technology (UST), Seoul 02792, South Korea.

**Keywords:** Artificial vision, retinal implant, retinal prosthesis, electrical stimulation, direction-selective RGC

## Abstract

To restore the sight of individuals blinded by outer retinal degeneration, numerous retinal prostheses have been developed. However, the performance of those implants is still hampered by some factors including the lack of comprehensive understanding of the electrically-evoked responses arising in various retinal ganglion cell (RGC) types. In this study, we characterized the electrically-evoked network-mediated responses (hereafter referred to as electric responses) of ON-OFF direction-selective (DS) RGCs in rabbit and mouse retinas for the first time. Interestingly, both species in common demonstrated strong negative correlations between spike counts of electric responses and direction selective indices (DSIs), suggesting electric stimulation activates inhibitory presynaptic neurons that suppress null direction responses for high direction tuning in their light responses. The DS cells of the two species showed several differences including different numbers of bursts. Also, spiking patterns were more heterogeneous across DS RGCs of rabbits than those of mice. The electric response magnitudes of rabbit DS cells showed positive and negative correlations with ON and OFF light response magnitudes to preferred direction motion, respectively. But the mouse DS cells showed positive correlations in both comparisons. Our Fano Factor (FF) and spike time tiling coefficient (STTC) analyses revealed that spiking consistencies across repeats were reduced in late electric responses in both species. Moreover, the response consistencies of DS RGCs were lower than those of non-DS RGCs. Our results indicate the species-dependent retinal circuits may result in different electric response features and therefore suggest a proper animal model may be crucial in prosthetic researches.

## I. Introduction

Outer degenerative retinal diseases, such as age-related macular degeneration (AMD) and retinitis pigmentosa (RP), are the leading causes of irreversible vision loss in Western countries [1], [2]. Those diseases cause gradual loss of photoreceptors that can lead to synaptic remodeling of the complex retinal circuitries [3], [4]. For those blinded by these ailments, retinal prostheses could be a promising option for vision restoration [5]–[14]. Multiple retinal prostheses showed impressive clinical outcomes by electrically stimulating remaining retinal neurons including bipolar cells and retinal ganglion cells (RGCs) [5]–[14]. A couple of the prosthetic devices (*i.e*., Argus II and Alpha-IMS/AMS) had become commercially available around the world. Also, PRIMA has recently shown great promise with reported visual acuity levels of 20/460 [13]. However, this level of visual acuity is still below the legal blindness (20/200) and far removed from the normal sight (20/20). Moreover, the recent work reported 7–8 seconds to identify letters [13], suggesting artificially-elicited neural signals may be less decipherable.

To create fast-recognizable and natural artificial vision, it would be essential to comprehensively understand electrically-evoked responses arising in RGCs [14], [15]. However, the remarkable complexity of the retina makes it extremely challenging. For example, there are numerous different types of RGCs [16]. It has been known each type encodes unique features from the visual world [17], [18], and the extracted information from all types is transmitted in parallel to the brain [19]. Among those, ON and OFF types which detect brightness increment and decrement have been studied in many previous works [20]–[29] because these two types are known to be critical for visual percepts [30], [31]. In addition to ON and OFF types, the mammalian retina has direction-selective (DS) types of RGCs to better encode the dynamic features [32]–[35]. In particular, ON-OFF subtype of DS RGCs accounts for nearly 20% of the whole RGC population in the mouse retina [16]. However, despite the crucial role and great portion, no study has investigated their network-mediated responses arising from indirect activation (*but see* [36], [37] for direct responses of DS RGCs).

Activating presynaptic neurons seem to be important for better-recognizable artificial vision because network-mediated responses can resemble each RGC’s own light-evoked responses at least in some types [14]. Our previous work also demonstrated differing levels of similarities between visually- and electrically-elicited responses in ON vs. OFF types, suggesting that certain retinal circuits may work better to produce natural responses [14]. Given the fact that presynaptic circuits of DS cells are known to be the most complicated in the retina, their network-mediated responses may be somewhat different from those of non-DS RGCs. Non-human primates are known to also have rabbit/mouse-like DS circuit elements and presynaptic inhibitory neurons such as starburst amacrine cells (SACs) [38]–[42]. Also, it has recently been reported that recursive bistratified cells in the primate retina are homologues of ON-OFF DS RGCs in the rabbit retina [43], [44]. Although the portion of those directionally sensitive cells in the whole RGC population has not been identified, it is of great interest to study network-mediated responses of DS cells for better prosthetic vision.

Here, for the first time, we recorded and systematically characterized network-mediated responses of ON-OFF DS RGCs (hereafter referred to as DS RGCs) from rabbits and mice which are the two most common animal species used in retinal studies. The use of the two different species enabled us to explore species-dependency in the correlation of light responses and electric responses of a given DS RGC as well as in several properties of network-mediated responses.

## II. Methods

### A. Preparation of Retina

We performed experiments under institutional and federal/national guidelines for animal use and care. Experiment protocols were approved by the Institutional Animal Care and Use Committees of Massachusetts General Hospital (2012N000111) and KIST (KIST-2020–156). New Zealand White rabbits (~2.5 kg) were anesthetized by intramuscular injections of a cocktail of xylazine/ketamine and euthanized with sodium pentobarbital intracardial injection. Wild-type mice (C57BL/6J) were anesthetized via inhalation of vaporizing isoflurane and euthanized by cervical dislocation. After the euthanasia, a retina tissue was extracted from an enucleated eyeball and flat-mounted on a filter paper, photoreceptor cell layer facing down. A small hole at the center of the filter paper (~2 mm in diameter) allowed the light illumination from the bottom side of the retinal tissue onto the photoreceptor outer segment layer.

### B. Electrophysiology

Patch electrodes (9–12 MΩ) filled with oxygenated Ames medium were used to remove the inner limiting membrane as well as to record spiking activities of RGCs in cell-attached mode. Two silver chloride-coated silver wires were used as the ground and located at the opposite edges of a recording chamber. Data were recorded and low-pass filtered at 2 kHz using an amplifier (Axopatch 200B or MultiClamp 700B, Molecular Devices, Sunnyvale, CA). Acquired data were digitized by a data acquisition card (PCI-MIO-16E-4, National Instruments, Austin, TX). Retinal samples were constantly perfused at 4 mL/min with oxygenated Ames medium which was maintained at 34–36°C.

### C. Light Stimulation for Cell Type Classification

Light stimuli were delivered to retinal samples by an LCD projector and a reflection mirror installed below the condenser of an upright microscope. Targeted RGCs were classified as ON-OFF type if they responded to both onset and offset of 1-sec-long stationary white spot flashes in diameters ranging from 100 to 1000 *μ*m on a gray background. Those RGCs were further tested with an elongated white bar on a gray background (width: 300 *μ*m, length: 1800 *μ*m, speed: 600 *μ*m/sec), which moved in 12 different directions (0–330° in 30° steps) [45]. Light stimuli were generated by custom scripts written in MATLAB (MathWorks, Natick, MA) and LabVIEW (National Instruments, Austin, TX). All visual stimuli were repeated at least 3 times. Timings of the elicited spikes were detected from raw recordings by software written in MATLAB.

### D. Computation of Direction Selectivity Indices

**Fig. 1.**
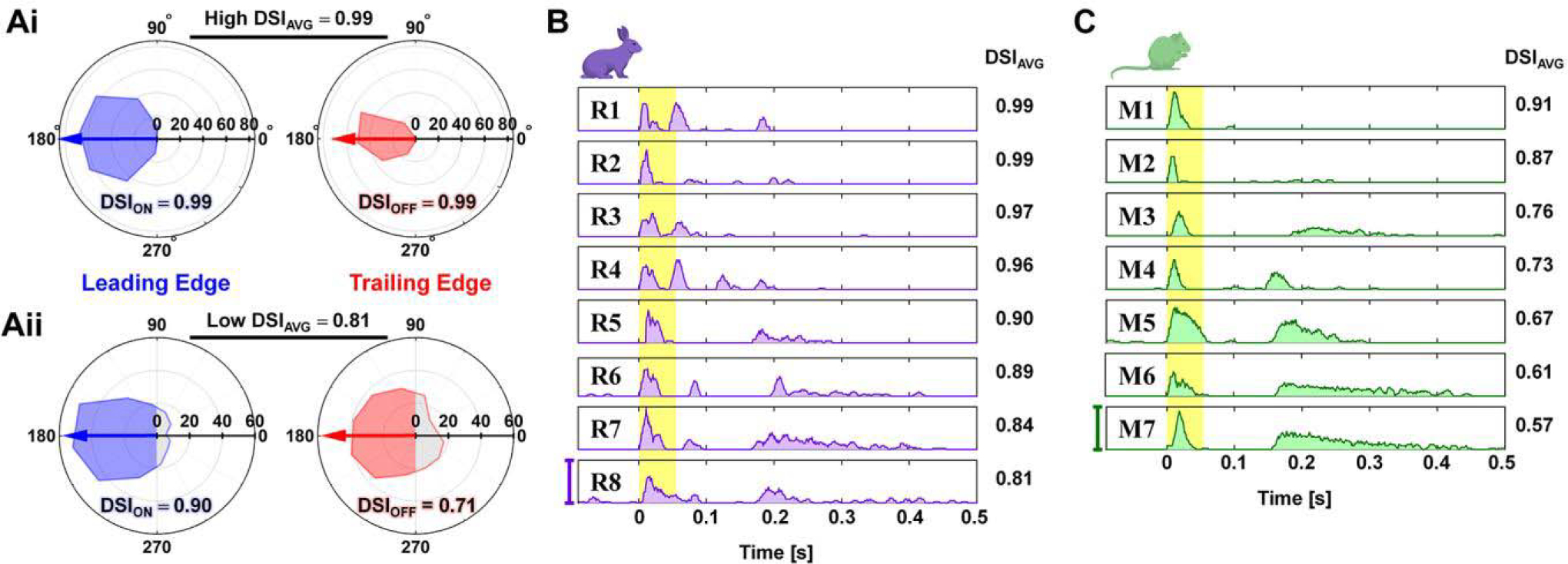
Electric responses of DS RGCs are heterogeneous. (Ai) Polar plots of light responses (spike count) to white moving bar stimulation from a highly directional DS RGC (DSI_AVG_ = 0.99). ON (leading edge) and OFF (trailing edge) responses are shown in blue and red, respectively. An arrow indicates the preferred direction vector (length is not to scale). (Aii) Same as *Ai* but for another DS RGC with low direction selectivity (DSI_AVG_ = 0.81). Area of preferred-side half (Area_Preferred_) is shown in blue or red while area of null-side half (Area_Null_) is shown in gray color. (B) Peristimulus time histogram (PSTH) of electric responses arising in rabbit DS RGCs; each row shows the average response of individual DS RGC to stimulation repeats (see Methods). The rows are arranged in the order of DSI from the highest to the lowest (right column shows DSI_AVG_ of each cell). Scale bar at bottom left (500 Hz) applies to all rows. A yellow vertical band indicates the time window of 55 ms from the stimulus onset, marking early responses. (C) Same as *B* but for responses of mouse DS RGCs.

ON-OFF RGCs showed robust spiking responses to both leading and trailing edges of the moving bar stimuli (hereafter referred to as ON and OFF responses, respectively). From the spike counts in responses to moving bars, polar plots were created for ON and OFF responses as shown with blue and red contours in Fig. 1A, respectively. Direction selectivity indices (DSIs) for ON and OFF responses of a given cell were calculated as follows [45]:

$$\text{DSI}=1-\frac{{\text{Area}}_{\text{Preferred}}}{{\text{Area}}_{\text{Null}}}$$

where Area_Preferred_ and Area_Null_ are the area of the preferred-side half and the other (*i.e*., null-side) half as shown with blue/red and gray polygons in each polar plot (Fig. 1Aii). The preferred direction was calculated as the vector sum of spiking responses for all 12 directions. Polar plots were rotated to have preferred directions at 180° and then Area_Preferred_ and Area_Null_ became the areas of left and right halves in each polar plot (Fig. 1Aii). The average of ON and OFF DSIs (DSI_AVG_; hereafter referred to as DSI) was used in our study; we excluded RGCs which had DSI < 0.5 to limit our study for highly directional cells. In total, we analyzed responses of 8 cells from 6 rabbits and 7 cells from 6 mice.

### E. Electric Stimulation

**Fig. 2.**
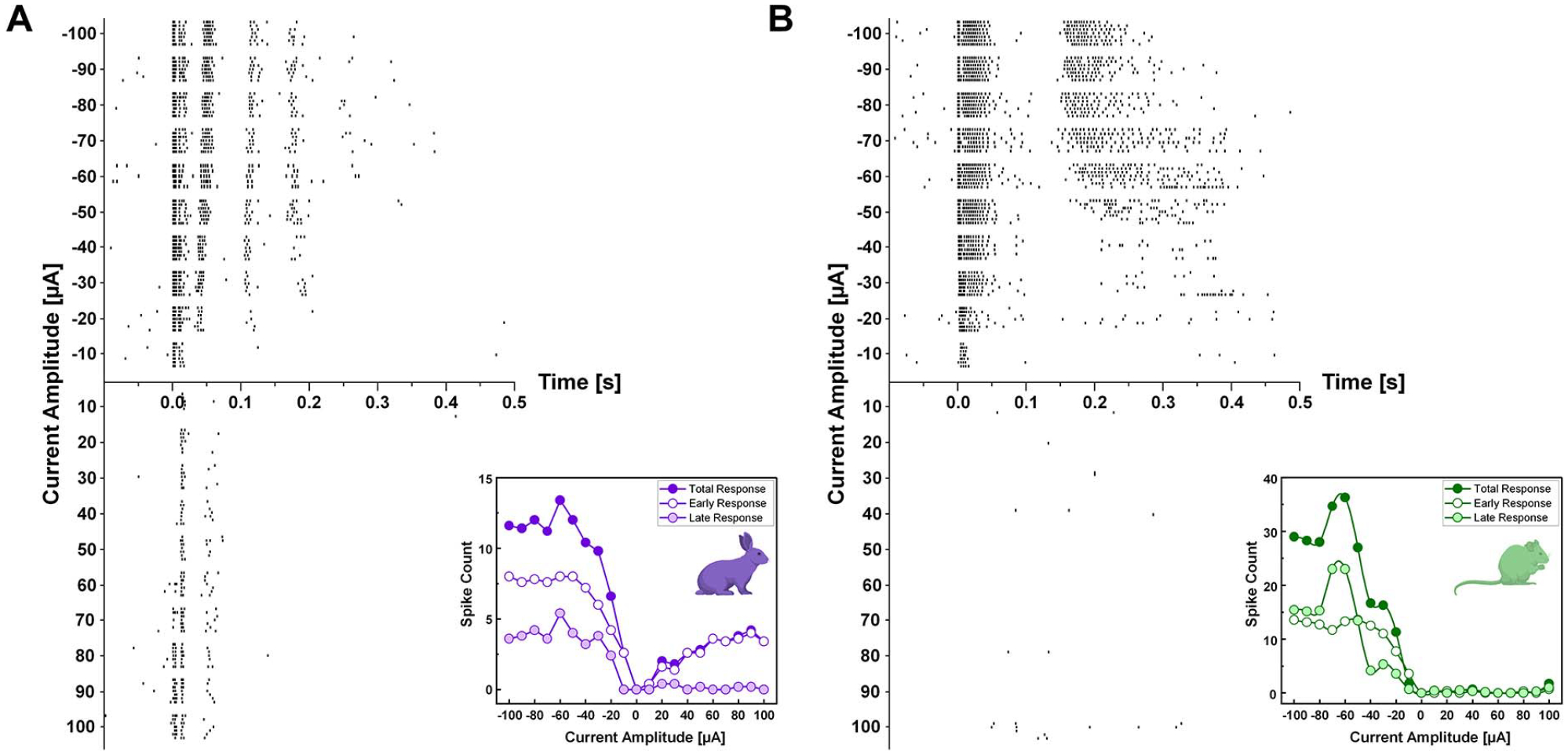
Electrically-evoked response patterns are similar across a wide range of current amplitudes in both species. (A) Raster responses arising in a rabbit DS RGC that corresponds to the 4th cell (R4) shown in Fig. 1B were plotted as a function of varying current amplitude, ranging from −100 to 100 *μ*A in 10 *μ*A steps. A minimum of five repeats were delivered at each amplitude. (B) Same as *A* but for responses of a mouse DS RGC that corresponds to the 5th cell (M5) shown in Fig. 1C. Insets in panels *A* and *B* showed spike count changes during total, early, and late response as a function of stimulation amplitude.

To deliver electric stimuli, we used commercial 10 kΩ platinum-iridium electrodes (MicroProbes, Gaithersburg, MD). The conical tip without insulation layer in each electrode had an approximate height of 125 *μ*m and a base diameter of 30 *μ*m, exposing a surface area of ~5900 *μ*m^2^. The stimulating electrode tip was controlled by a micro-manipulator and located ~25 *μ*m above the inner limiting membrane. Electric stimulus was a 4-ms-long monophasic current, typically −100 *μ*A in all cases but Fig. 2 which tested a broad range of current amplitudes. This stimulus condition (*i.e*., duration and current amplitude) evoked strong network-mediated responses in non-DS RGCs by activating presynaptic neurons [14]. Although the very first spike of the elicited response is known to be direct activation [14], we refer whole responses as network-mediated because all other subsequent spikes are resulted from activated presynaptic neurons. The electric stimuli were generated by a stimulus generator (STG2004, Multi-Channel Systems GmbH, Reutlingen, Germany) and controlled by custom software written in LabVIEW and MATLAB. An identical electric stimulus was repeated at least 5 times (typically 7 times) for a given cell; a recovery time (>2 seconds) was allowed between successive stimuli.

### F. Analyses of Electric Responses

**Fig. 3.**
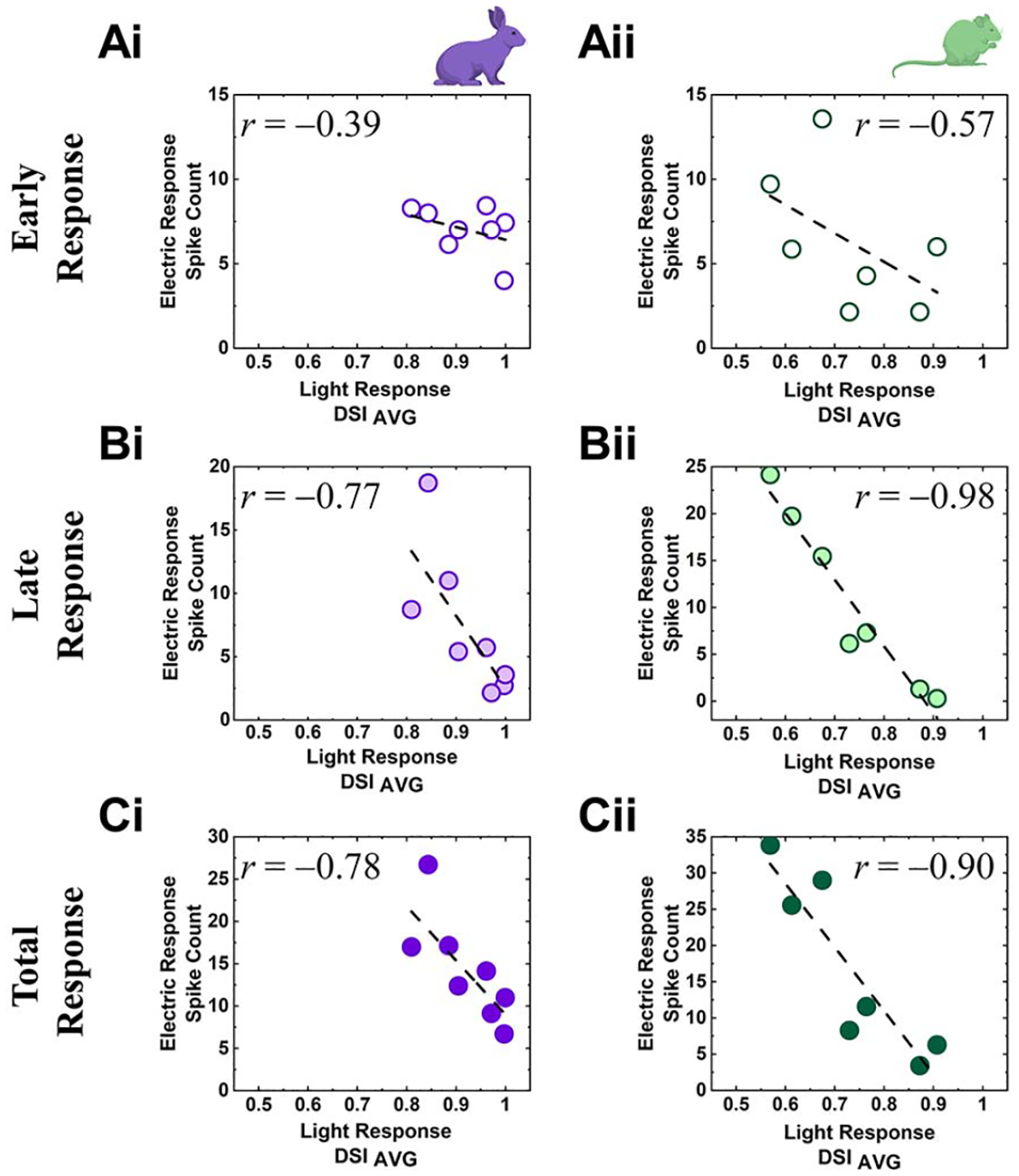
Electric responses of DS RGCs are inversely correlated with their DSIs. (Ai) Scatter plot of spike count during early response vs. light response DSIs of rabbit DS RGCs. A dashed line indicates the linear fitting curve and the level of correlation (Pearson’s *r* value) is shown in the plot. (Bi-Ci) Same as *Ai* but for late and total responses, respectively. (Aii-Cii) Same as *Ai*-*Ci* but for mouse DS RGCs.

**Fig. 4.**
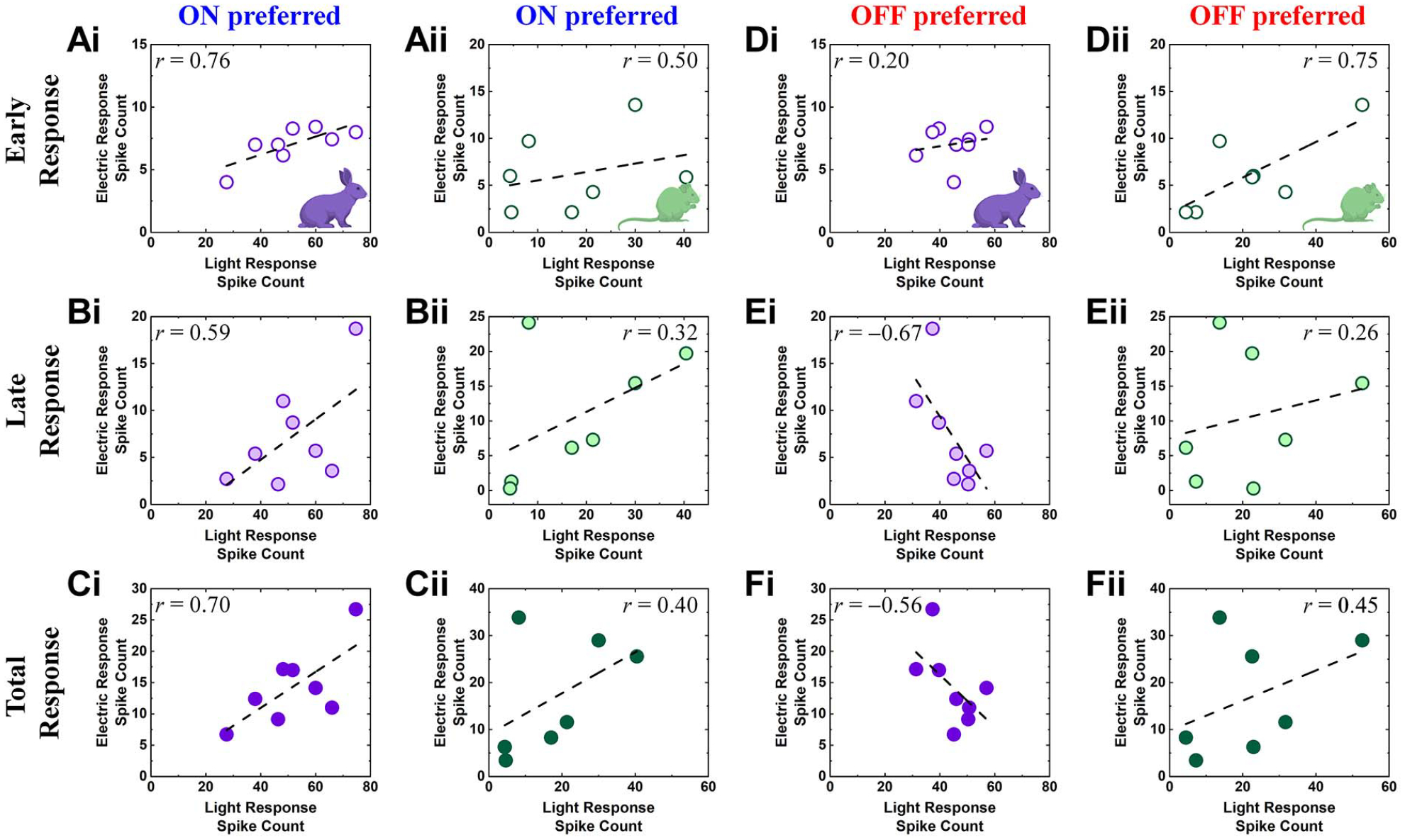
Electric response spike counts show positive correlation with light response spike counts in all but OFF preferred light responses of rabbit DS RGCs. (Ai-Ci) Scatter plots of electric response (spike count) vs. ON light response (spike count) in the same cell for all DS RGCs in the rabbit retina for early (*Ai*), late (*Bi*), and total (*Ci*) responses, respectively. A dashed line indicates the linear fitting curve and the level of correlation (Pearson’s *r* value) is shown in the plot. (Aii-Cii) Same as *Ai*-*Ci* but for mouse DS RGCs. (Di-Fi) Same as *Ai-Ci* but for OFF light responses. (Dii-Fii) Same as *Di*-*Fi* but for mouse DS RGCs.

Electrically-evoked spike timings were detected by custom MATLAB scripts that also removed electric artifacts from raw recordings. Spiking activities were represented in forms of peristimulus time histograms (PSTHs; Figs. 1B and 1C) and raster plots (Fig. 2). In raster plots, each vertical tick represents a single spike. For PSTHs, we computed firing rates in each 10-ms-long bin with a rolling step of 1 ms. In each DS RGC, spiking activities were divided into early and late responses depending on post stimulus latencies (0–0.055 s and 0.055–0.500 s for early and late responses, respectively). This separation was made because the longest duration of the early burst with firing rate above 50 Hz was 51 ms and was separated by >~9 ms from any subsequent firing response. Then, we correlated each component of electrically-evoked responses (*i.e*., early, late, and total responses) of each RGC with properties of their own light-evoked responses (*i.e*., DSIs, ON or OFF responses to bars moving in preferred direction) in scatter plots (Figs. 3 and 4).

**Fig. 5.**
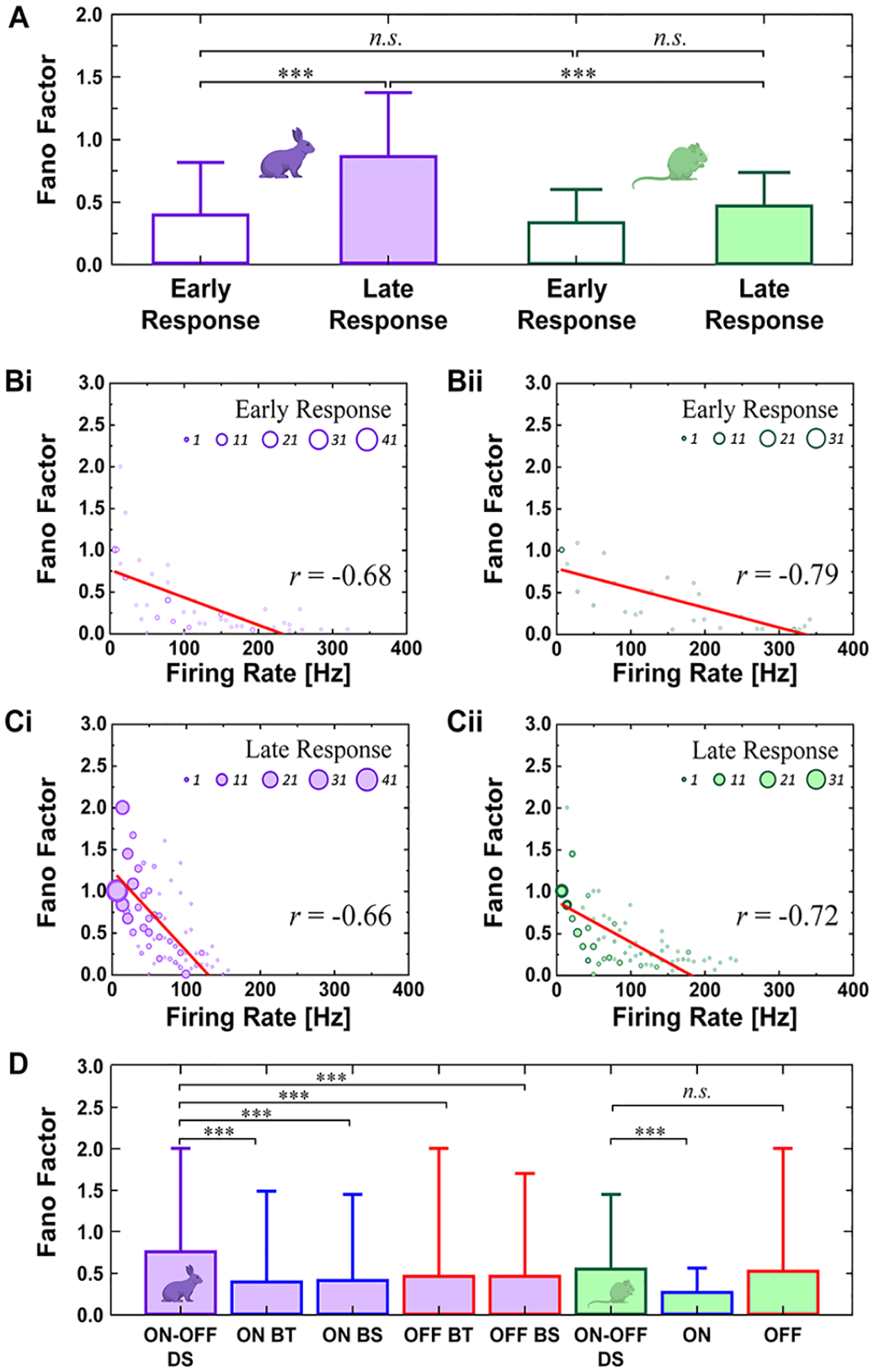
DS RGCs show low inter-trial consistency of spike counts across repeats of electric stimulation. (A) Average Fano Factors (FFs) of early and late responses in rabbit and mouse retinas. FFs were calculated for every 20-ms-long bin with a step of 5 ms. Bars represent means and error bars indicate SDs. Statistical significance was assessed using the one-way ANOVA with Holm-Sidak post-hoc comparisons; ***p < 0.001 and n.s. means not significant. (B) FFs were plotted as a function of firing rate of each bin for early responses of DS RGCs in rabbit (*Bi*) and mouse (*Bii*) retinas. Size of circle indicates number of bins at each data point (see legend) (C) Same as *B* but for late responses in rabbit (*Ci*) and mouse (*Cii*) retinas. (D) FFs of total responses were compared across DS RGCs and non-DS RGCs of both species. Bar graphs are shown in purple and green for rabbit and mouse RGCs. Statistical significance was assessed in a given specie.

**Fig. 6.**
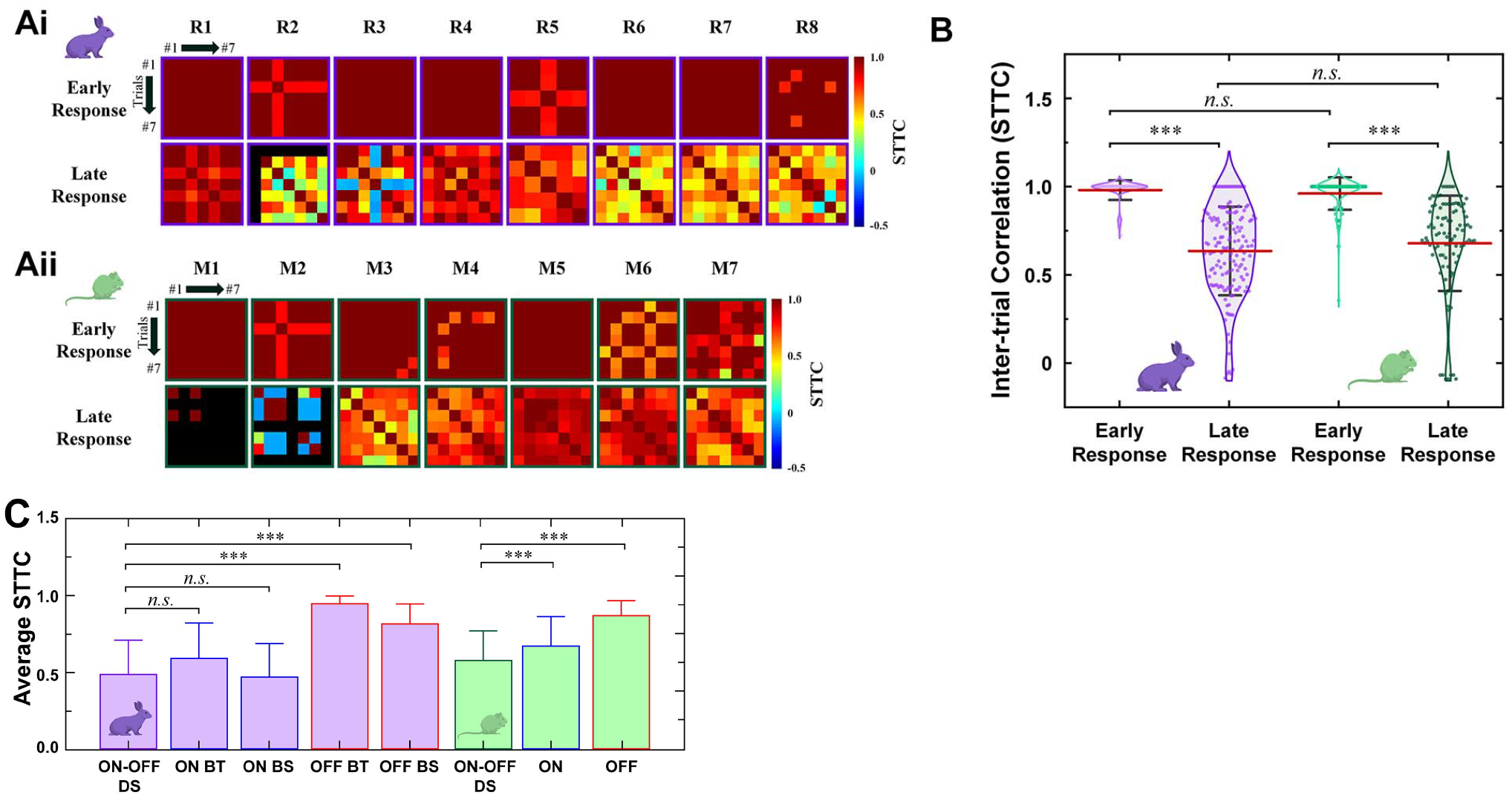
Spike timing consistency is lower in DS RGCs than non-DS RGCs. (A) Color-coded heatmaps of the spike time tiling coefficients (STTCs) of early and late responses for each DS RGC from rabbit (*Ai*) and mouse (*Aii*) retinas. An identical stimulus repeated for 7 times for all cells but R5 which had 5 repeats. Black color in matrices of M1 and M2 indicates no late response was elicited in those trials. (B) Violin plots of all STTC values computed in each DS RGC. (C) Comparisons of average STTCs of DS vs. non-DS RGCs in rabbit (purple-filled bars) and mouse (greed-filled bars). Bars represent mean and errors bars indicate SD. Statistical significance was assessed only within a given species. Every possible pair was tested; following pairs also showed statistical significance but are not shown for brevity: ON BT vs. ON BS and OFF BT vs. OFF BS of rabbit retinas, and ON vs. OFF of mouse retinas.

We also assessed electric response consistencies in a given cell. First, we examined spike count consistency across repeats of electric stimulation by computing Fano Factor (FF), which is the ratio of the variance to the mean of spike counts [46]. For early and late responses, FFs were averaged across DS RGCs (Fig. 5A). Also, FFs were calculated in all 20-ms-long bins which were moved in a step of 5 ms [29] and plotted as a function of the firing rate of each bin (Figs. 5B and 5C). Second, we examined spike timing consistency by computing spike time tiling coefficient (STTC) across repeats [47]:

$$\text{STTC}=\frac{1}{2}\left(\frac{P_{A}-T_{B}}{1-P_{A}T_{B}}+\frac{P_{B}-T_{A}}{1-P_{B}T_{A}}\right)$$

where *P*_*A*_ is the proportion of spikes from A that lie within ± time window (±Δt) of each spike from the spike train ***B*** (*P*_B_ calculated similarly), *T*_A_ is the proportion of total recording time which has any spikes within ±Δt of any spike from the spike train ***A*** (*T*_B_ calculated similarly). We used Δt of 10 ms in this work. The STTC represents the correlation level of two different spike trains by comparing the similarity of spike timings [47]. To display the spike timing variability across repeats in each cell, STTCs of early and late responses were plotted in a form of heat matrices (Fig. 6A). Also, average STTC values in both responses of all DS RGCs were shown as violin plots for each species (Fig. 6B). For total responses (*i.e*., early and late responses together), FFs and STTCs were computed and compared with those of non-DS RGCs (Figs. 5D and 6C), which were retrospective data from our previous works using rabbit [14] and mouse [29] retinas, respectively.

### G. Statistical Analysis

Unless otherwise indicated, all data were expressed as the mean ± standard deviation (SD). Statistical analysis was per formed using a one-way ANOVA with Holm-Sidak post-hoc comparisons; *p* < 0.05 was considered statistically significant. In scatter plots, correlations were evaluated using Pearson’s product-moment coefficient (*i.e*., Pearson’s *r*).

## III. Results

### A. Network-Mediated Responses of ON-OFF DS RGCs Are More Heterogeneous Than Non-DS ON or OFF RGCs

ON-OFF DS RGCs (hereafter referred to as DS RGCs) are known to evoke a robust burst response to elongated bars of light moving in preferred direction while weak or almost no spikes to bars moving in the opposite (null) direction [16], [32]–[36], [45]. When the leading/trailing edge of a white moving bar entered/exited the receptive field of a given DS RGC, ON/OFF responses were elicited, respectively. As illustrated in polar plots (Figs. 1Ai and 1Aii), DS RGCs showed asymmetric ON and OFF response magnitudes depending on the direction of moving bars. From these polar plots, we computed and averaged DSIs (*see* METHODS) to represent the level of directional tuning of each cell. In case of the DS RGC which showed no spike in response to the null direction, both ON and OFF responses resulted in high DSIs (Fig. 1Ai). On the other hand, a substantial presence of null direction responses resulted in much lower DSIs (Fig. 1Aii).

We show the PSTHs of electric responses of all DS RGCs in the descending order of their DSIs (Figs. 1B and 1C). The spiking patterns of DS RGCs appeared to be heterogeneous across the two species as well as across cells in each species. For example, the rabbit DS RGCs responded with distinctly different numbers of spike bursts and inconsistent timing of those bursts even with similar DSIs (*compare* R1 vs. R2, R3 vs. R4, and R5 vs. R6 in Fig. 1B). In the mouse retina, however, the first two DS RGCs evoked a single burst of spikes whereas the rest of the cells evoked another burst that was separated by a long spike-free period (Fig. 1C). Earlier, it had been thought that the same type of RGCs receives synaptic input from identical synaptic circuitries [18] and therefore their electrically-evoked responses would be similar. Indeed, our previous study reported that networked-mediated responses of ON or OFF RGCs were unique across subtypes but similar in a given type [14]. Also, those electric responses were similar in homologue RGC types of rabbit and mouse retinas [14], [25]–[29], [48]. On the contrary, the electric responses of DS RGCs seemed to be quite different across those two species (Figs. 1B and 1C).

### B. Network-Mediated Response Patterns Were Similar Across a Wide Range of Stimulation Amplitude

In clinical trials, various amplitudes of electric stimulation are used for brightness modulation [49]. Our earlier study showed that network-mediated responses of non-DS RGCs are systematically modulated by varying current amplitudes [14]. To test whether this was the case in DS RGCs as well, we applied a wide range of electrical pulses from −100 to 100 *μ*A with the same pulse duration (Figs. 2A and 2B for rabbit and mouse, respectively). Generally, in both species, responses to cathodal stimuli got stronger as the current amplitude increased (upper halves of Figs. 2A and 2B). Consistent with those of non-DS RGC types [14], early and late responses of DS RGCs were saturated around −50 or −80 *μ*A (*insets* of Fig. 2). Thus, in following analyses, we used responses of DS RGCs arising from −100 *μ*A current stimulus, which showed well saturated spike counts.

In case of anodal stimuli, the rabbit DS RGC exhibited bigger responses with increasing current amplitude while the mouse DS RGC evoked almost no response to the full range of stimulus amplitudes (lower halves of Figs. 2A and 2B). The robust anodal responses observed in the rabbit cell (Fig. 2A) are consistent with those of brisk transient (BT) subtypes of ON or OFF RGCs [14]. On the other hand, the response patterns of the mouse DS RGC to both cathodal and anodal stimuli (*i.e*., two bursts of spikes and almost no spike, respectively; Fig. 2B) are similar to what was previously found from the sustained subtype of alpha ON RGCs in the mouse retina [28]. Although the sample size was limited (n = 1 for each species) due to the time-consuming nature of recording for wide ranges, these two examples suggest that electric responses of DS RGCs may have properties of both transient and sustained pathways (*see* DISCUSSION).

### C. Electric Response Magnitudes Were Inversely Proportional to DSIs

The earlier PSTHs showed smaller response magnitudes as DSIs increased (Figs. 1B and 1C). We further examined the correlation between response magnitudes and DSIs by plotting spike counts of early, late, and total responses as a function of DSI (Fig. 3). Negative correlations were found in every case, indicating high directional tuning reduced electric responses. Although the early responses of both species showed weak correlations (Figs. 3Ai and 3Aii), the levels of the correlation were much stronger in the late responses (Figs. 3Bi and 3Bii). Consistent with our previous report [25], the results shown here also suggest that inhibitory neurons are likely to be critical in determining electric response patterns. For example, starburst amacrine cells (SACs) are known to be a key neuronal element in the DS circuit by inhibiting null-direction responses [32], [40]-[42].

### D. Correlation Trends Between Electric Response and Light Response Were Different in Rabbit and Mouse DS RGCs

Prosthetic vision is likely to be improved if electric responses of each RGCs better resemble the responses that arise naturally to light [14], [15], [50]. Therefore, it is important to compare light and electric responses in each cell. We compared spike counts of light vs. electric responses (Fig. 4) with preferred direction moving bar responses as light responses; leading and trailing edge responses were both compared to see if any difference between ON and OFF channels exists. In both rabbits and mice, early, late, and total responses arising from a cathodal stimulus (~100 *μ*A) in DS RGCs (electrical) were all well correlated with the same-cell light responses (white bars moving in the preferred direction) (Figs. 4Ai–4Ci and Figs. 4Aii–4Cii). The correlation degree was maximal during early responses in both species (Figs. 4Ai and 4Aii). In sharp contrast, rabbit DS RGCs resulted in a negative correlation between OFF light responses and late responses with fairly high *r*-value (*r* = −0.67; Fig. 4Ei). On the other hand, in the mouse DS RGCs, the correlation trends between OFF light responses and electric responses were largely similar to those between ON light responses and electric responses (*compare* Figs. 4Dii–4Fii with Figs. 4Aii–Cii). The correlation between OFF light responses and early electric responses became more positive (Fig. 4Dii) compared to ON light responses (Fig. 4Aii). Although OFF light responses and early responses of the rabbit DS RGCs seem to have positive correlation, the correlation level was much smaller than those of the mouse DS RGCs (compare Figs. 4Di and 4Dii). These results suggest electric stimulation may trigger presynaptic networks of DS RGCs in different manners between rabbit and mouse retinas, particularly in the OFF pathways.

### E. Spike Count Consistency of Network-Mediated Responses Was Lower in DS RGCs Than Non-DS RGCs

For prosthetic users at a fixed gaze, the high consistency of electric responses across repeats may improve the recognition of artificial visual percepts [29]. It is because the reliability of neuronal responses seems to be important in the visual system [51] and reliable behavioral responses [52]. To measure spike count consistencies of the electric responses across repeats, we first computed Fano Factors (FFs) for early and late responses of each cell (*see* METHODS). There were distinct disparities between the species: in the rabbit DS RGCs, the average FF of late response (0.85 ± 0.51) was substantially bigger than that of the early response (0.38 ± 0.42) (Fig. 5A), which is consistent with our previous report with non-DS cells [29]. On the other hand, in the mouse DS RGCs, the average FF of late responses (0.55 ± 0.37) was not considerably different (*i.e*., no statistical significance) from that of early responses (0.41 ± 0.35). Actually, the average FFs of early responses in both species were much bigger than those of non-DS ON and OFF cells of wild-type mice (0.12 ± 0.14 and 0.03 ± 0.06) [29], clearly indicating inferior spiking consistencies of DS cells even from early responses.

Having the higher average FFs in late responses, the rabbit DS RGCs seemed to be worse at reproducing a consistent number of spikes than the mouse DS RGCs. Reason of this contrast was found in the scatter plots of FFs that were computed in every 20-ms-long bin in spiking responses (Figs. 5B and 5C). Our plots revealed the rabbit DS RGCs generated spiking responses with more low-firing bins, resulting in the bigger average FF due to the inverse correlation between FFs and firing rates [29], [46].

We also compared the average FFs of total responses as a whole (*i.e*., without distinguishing early or late component) across DS vs. non-DS types. For the comparison, we used our old data sets of non-DS RGCs: 19 ON brisk transient (BT), 22 ON brisk sustained (BS), 16 OFF BT, and 23 OFF BS cells from rabbit retinas [14]; 6 ON and 7 OFF cells from mouse retinas [29]. The rabbit DS RGCs showed higher FFs (0.75 ± 0.53) with statistical significance than every non-DS type (0.39 ± 0.42, 0.41 ± 0.35, 0.46 ± 0.51, and 0.45 ± 0.43 for ON BT, ON BS, OFF BT, and OFF BS, respectively; Fig. 5D). However, the average FF of the mouse DS RGCs (0.54 ± 0.38) was similar to that of non-DS OFF cells (0.26 ± 0.23) but slightly bigger than that of ON cells (0.52 ± 0.54).

### F. Spike Timing Consistency of Network-Mediated Responses Was Lower in DS RGCs Than in Non-DS RGCs

For reliable prosthetic visual percepts across stimulation repeats, spike timing may be also crucial [29]. To measure the spike timing consistency, we computed spike time tiling coefficients (STTCs) from responses of each cell to multiple repeats (*see* METHODS) and created correlation matrices (Fig. 6A). In general, spike timings of the early responses were largely consistent across repeats in any given cell in both rabbit and mouse retinas (first rows of Figs. 6Ai and 6Aii). However, the late responses showed much decreased STTCs, indicating that their spike timings became more variable across repeats of stimulation (second rows of Figs. 6Ai and 6Aii). As shown in the scatter violin plots of STTCs (Fig. 6B), the average STTC value was significantly reduced in late than in early responses for both species. However, there was no statistical significance between early responses of the two species (0.98 ± 0.06 vs. 0.96 ± 0.09 for rabbit vs. mouse DS RGCs, respectively; red bars in Fig. 6B). Also, no statistical difference was horizontal shown between late responses (0.64 ± 0.25 vs. 0.68 ± 0.27 for rabbits and mice).

The average STTCs of total responses were also compared across DS vs. non-DS RGCs using the same old data sets which were used in Fig. 5D. In the rabbit retinas, electric responses of DS RGCs showed the average STTC (0.49 ± 0.23) similar to those of non-DS ON cells (0.59 ± 0.23 and 0.47 ± 0.22 for BT and BS subtypes, respectively; no statistical significance; Fig. 6C). In contrast, the non-DS OFF cells showed much higher average STTCs (0.95 ± 0.05 and 0.82 ± 0.13 for BT and BS subtypes, respectively; *compare* purple bars of Fig. 6C) than that of DS RGCs. This is because both subtypes of OFF RGCs generated highly consistent response patterns across repeats [14], [48]. On the other hand, in the mouse retinas, the average STTC of DS RGCs (0.58 ± 0.20) was lower than those of both ON and OFF RGCs (0.67 ± 0.20 and 0.87 ± 0.10, respectively; *compare* green bars of Fig. 6C). Taken together, we can conclude that consistencies of network-mediated electric responses are lower in DS RGCs than in non-DS RGCs for both species.

## IV. Discussion

### A. Network-Mediated Responses Arising From Electric Stimulation Are More Heterogeneous in DS RGCs Than in Non-DS RGCs Between Species as Well as Across Cells

In a previous study, we reported spiking response patterns arising in a given type of non-DS RGCs were largely similar in terms of number of bursts and latencies of those burst onsets [14]. This was somewhat expected as presynaptic neuronal circuits of each type of RGCs are known to be unique but generally similar across RGCs in a given type [18]. Thus, upon electric stimulation, we had expected to observe another unique spiking response patterns in DS RGCs. Moreover, we thought their response patterns would be quite similar between the two species we investigated because both ON and OFF types responded similarly to electric stimulation between rabbit and mouse retinas [14], [27]–[29], [48].

However, the overall variation of electric responses across DS RGCs of the two species was greater than that of non-DS RGCs. For instance, we found highly heterogeneous spiking responses between the two species as well as across the cells of a given species (Figs. 1B and 1C). Particularly, response patterns of each rabbit DS RGC were almost individually unique in burst count and latency across all the cells that we tested in this study. In the mouse DS RGCs, except the first two cells with high DSIs, the spiking responses typically consisted of two separate bursts (Fig. 1C), which showed contrasting difference from those of rabbits. Given the second burst latency, the response pattern of the mouse DS RGCs was similar to that of sustained type of ON alpha RGCs in the mouse retina [28].

**Fig. 7.**
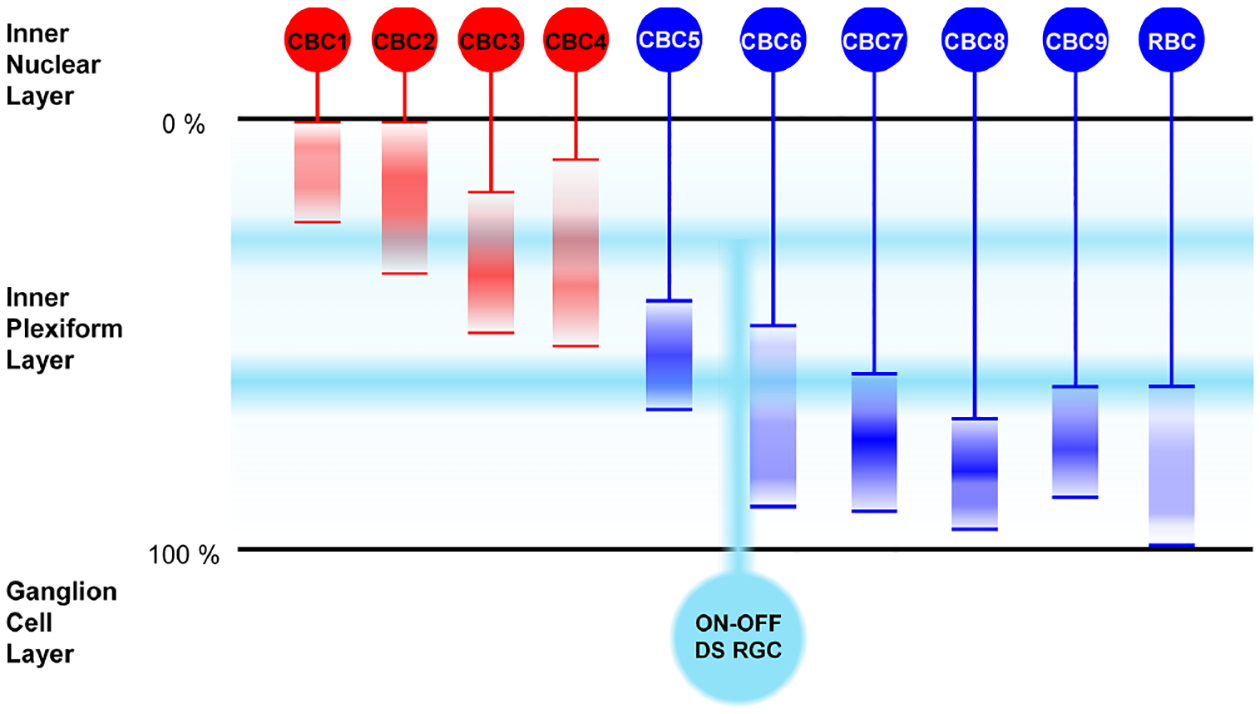
ON-OFF DS RGCs receive inputs from virtually all types of bipolar cells. Axon terminal depths of various types of cone bipolar cells (CBCs) and rod bipolar cell (RBC) are shown in red and blue vertical bands in the inner plexiform layer (IPL). OFF and ON types of bipolar cells are shown in red and blue color, respectively. Dendritic stratification depths of ON-OFF DS RGC are shown with two horizontal light blue shades in both ON and OFF sublaminae. Estimated stratifications depths are drawn based on the information published in [58]–[60].

These heterogeneous response patterns of DS RGCs may arise from the remarkable complexities of DS retinal circuits [32], [53]–[57]. First, ON-OFF DS RGCs are known to stratify their dendrites at both ON and OFF sublaminae of the inner plexiform layer (IPL) of the retina [16], [57]. Because those stratification depths are in the middle of the two sublaminae, ON-OFF DS RGCs receive inputs from virtually all different types of bipolar cells (BCs) (Fig. 7; [58]–[60]). Second, other classes of retinal neurons such as SACs and wide field ACs are involved in DS computations [41], [42], [45], [57]–[60]. Taken together, these anatomical characteristics of DS RGCs suggest a possibility that each DS RGC may receive inputs from those presynaptic neurons (*i.e*., diverse types of BCs and/or different numbers of SACs), depending on their directional tuning and preferred/null-direction response magnitude. Therefore, highly heterogeneous spiking activities were elicited by electric stimulation, and it might have been mediated by the temporally-unselective activation of the complicated presynaptic neurons of the DS RGCs. Consequently, it may be more reasonable to have more heterogeneous spiking patterns in network-mediated responses of DS RGCs compared to those of non-DS cells.

Also, DS circuits of the rabbit and mouse seem to diverge differently to meet their behavioral preference [61]. For instance, nocturnal or diurnal activities may cause different contrast sensitivities in the two species [62], [63]. As another example, Ding *et al*. also reported the difference in inhibitory synaptic connection of the DS circuit in the two species [61]. To be concise, AC input is restricted to the initial third of the dendritic trees in mouse SACs while the same synapses are formed at their distal dendrites in the rabbit SACs [64]–[67].

### B. Network-Mediated Response Magnitudes of DS RGCs Were Reduced by Increased Directional Tuning

SACs, the key component of DS circuit, strongly prefer centrifugal (toward the end of the dendrites) motion rather than centripetal (toward the soma), thus enabling the release of inhibitory signals asymmetrically [41]. Sharp direction tuning capabilities of DS RGCs are mostly mediated by SACs [32]: higher DSI cells are likely to receive more inhibitory inputs in response to visual stimuli of null-direction motion. Our experimental results suggest that the electric stimulus we used might have activated these inhibitory networks, resulting in systematically reduced responses in DS RGCs that showed higher directional tuning in both species (Fig. 3). Given that the bigger DSI requires less null-direction responses (Figs. 1Ai vs. 1Aii), DS RGCs with higher directionality might have received more inhibition from activated SACs and evoked smaller responses. Also, species-dependent dendritic locations of SACs in the rabbit and mouse retinas [61] may explain the different response patterns of the two species (Figs. 1B and 1C). On the other hand, DS RGCs with lower DSIs elicited relatively stronger spiking by the same electric stimulation because they might be surrounded by a smaller number of SACs. It is also worth noting that late responses showed more negative correlations, somewhat consistent with previous report of slow activation of amacrine cells after electric stimulation [68]. Although we did not study the underlying mechanism, further experiments with synaptic blockers are likely to offer new insights about how the inhibitory network shapes electrically-evoked responses, which may be useful for improved artificial vision.

### C. Late Electric Response Consistency Was Lower in DS Than Non-DS RGCs

It is important to evoke consistent spiking responses for eliciting the sustained artificial percept by retinal implants. In the healthy retina, non-DS RGCs generated pretty reliable spiking activities across repeats of the same electric stimulation [14]. However, this response reliability was decreased in the degenerate retina due to the loss of photoreceptors and synaptic changes [29]. However, a large portion of RGC types is still unstudied regarding their electric response reliability even in the healthy retinas. In that sense, we assessed the spike count and timing consistencies of each DS RGCs by computing FFs and STTCs. In common, the electric response spike count became more variable across repeats in the late responses due to the decreased firing rate (Figs. 5Bi–5Cii). The average FF of rabbit DS RGCs in the late responses was significantly higher than that of early responses; however, the FFs of the mouse DS RGCs showed no statistical significance between their early and late responses (Fig. 5A). These results indicate DS RGCs of rabbits were inferior to those of mice in evoking a consistent number of spikes across electric stimulation repeats.

Spike timing-wise, early response STTCs were quite close to 1, meaning that the early response spike timing was consistent across stimulation repeats. In the late responses, however, the response timing became more inconsistent as shown with low STTCs (Figs. 6Ai–6Aii). Interestingly, as we compared the average STTCs of DS RGCs and non-DS RGCs, the rabbit DS RGCs had statistical significance with OFF RGCs but not with ON RGCs (Fig. 6C). In contrast, the average STTCs of the mouse DS RGCs showed statistically significant difference with both ON and OFF types (Fig. 6C).

### D. Implication of the Present Study

Given the wide heterogeneity in the population of RGCs, deeper understanding of the electrically-evoked responses of diverse types of RGCs may enhance the quality of prosthetic vision. Also, because electric stimulation activates diverse types of RGCs in an indiscriminate way, thorough understanding of responses patterns of those various type would be of high importance. Between direct and indirect activation, it seems like the later which evoke network-mediated responses may have several benefits. For example, indirect activation can produce natural spiking responses which may be more recognizable [14]. Also, indirect activation may result in high level of cell-to-cell spiking heterogeneities for efficient neural information transmission [48] at least in the healthy retina. To improve our comprehensive understanding, in the present study, we characterized the network-mediated responses of DS RGCs which play an integral role in seeing the dynamic visual world by encoding the motion information [32]–[35].

The present study has a clear limitation that responses of DS RGCs were recorded from healthy animals. However, our experimental findings may have several implications. First, our study uncovered that DS RGCs of rabbits and mice generate differing electric responses due to the differences between DS circuits of the two species [61], [64]–[67]. Given that the subtle circuit differences can make contrasts in electric responses, our results suggest future research should use an animal model closer to humans, e.g., non-human primates (NHP) [69] for which the retinal circuits most closely resemble humans. A recent study reported midget and parasol cells in the human retina are not directional [70]. But, recursive bistratified cells of the NHP retina are known to be ON-OFF directionally selective [43] although they may have been identified owing to the paucity.

Second, it is highly likely that electric stimulation lacks the strong directional preference which is the defining hallmark of DS RGCs because of the non-selective activation property of electric stimulation. Thus, when having spiking activities from non-DS and DS cells all together, it seems like indiscriminate motion information of DS RGCs interfere with normal static percepts of artificial vision. Also, for conveying natural motion information, suppressing null direction responses seem to be a key for high direction tuning (i.e., high DSI) (Fig. 1A). Although both activating SACs and inhibiting DS RGCs would be challenging due to the presence of non-DS RGCs nearby, stimulation strategies should be optimized for the suppression of null responses.
